# Gestational intermittent hypoxia reduces mandibular growth with decreased *Sox9* expression and increased *Hif1a* expression in male offspring rats

**DOI:** 10.3389/fphys.2024.1397262

**Published:** 2024-06-11

**Authors:** Takumi Suzuki, Jun Hosomichi, Hideyuki Maeda, Yuji Ishida, Risa Usumi-Fujita, Manaka Moro, Korkuan Jariyatheerawong, Takashi Ono

**Affiliations:** ^1^ Department of Orthodontic Science, Graduate School of Medical and Dental Sciences, Tokyo Medical and Dental University (TMDU), Tokyo, Japan; ^2^ Department of Forensic Medicine, Graduate School of Medicine, Tokyo Medical University, Tokyo, Japan; ^3^ Department of Legal Medicine, Graduate School of Medicine, Osaka University, Osaka, Japan; ^4^ Department of Orthodontics, Faculty of Dentistry, Chulalongkorn University, Bangkok, Thailand

**Keywords:** obstructive sleep apnea, intermittent hypoxia, pregnant, DOHAD, skeletal growth, SOX9, HIF-1α

## Abstract

**Introduction:**

Maternal obstructive sleep apnea (OSA) during pregnancy is the risk factor for impaired fetal growth with low birth weight in the offspring. However, it is unclear whether gestational intermittent hypoxia (IH, a hallmark of maternal OSA) has long-term detrimental consequences on the skeletal development of offspring. This study aimed to investigate postnatal maxillofacial bone growth and cartilage metabolism in male and female offspring that were exposed to gestational IH.

**Methods:**

Mother rats underwent IH at 20 cycles/h (nadir, 4% O_2_; peak, 21% O_2_; 0% CO_2_) for 8 h per day during gestational days (GD) 7–20, and their male and female offspring were analyzed postnatally at 5 and 10 weeks of age. All male and female offspring were born and raised under normoxic conditions.

**Results:**

There was no significant difference in whole-body weight and tibial length between the IH male/female offspring and their control counterparts. In contrast, the mandibular condylar length was significantly shorter in the IH male offspring than in the control male offspring at 5 and 10 weeks of age, while there was no significant difference in the female offspring. Real-time polymerase chain reaction (PCR) showed that gestational IH significantly downregulated the mRNA level of SOX9 (a chondrogenesis marker) and upregulated the mRNA level of HIF-1α (a hypoxia-inducible factor marker) in the mandibular condylar cartilage of male offspring, but not in female offspring.

**Conclusion:**

Gestational IH induced underdeveloped mandibular ramus/condyles and reduced mRNA expression of SOX9, while enhancing mRNA expression of HIF-1α in a sex-dependent manner.

## 1 Introduction

Fetal development is susceptible to maternal exposure to environmental stressors such as hypoxia, metabolic stress, and malnutrition (Nalivaeva et al., 2018; Ren et al., 2023). Obstructive sleep apnea (OSA) is a sleep disorder with a prevalence of 10.5% and 26.7% in pregnant women in the early and late stages of pregnancy, respectively (Pien et al., 2013). The concept that environmental stressors during fetal development influence the organism’s adaptation to conditions later in postnatal life is known as the “Developmental Origins of Health and Disease” (DOHaD) paradigm (Heindel et al., 2015). Maternal OSA affects the impaired fetal growth with low birth weight in the offspring and increases the risk of cardiovascular and neurodevelopmental disorders in offspring (Fung et al., 2013; Chen et al., 2018; Vanderplow et al., 2022). However, it is unclear whether gestational IH (a hallmark of maternal OSA) has long-term detrimental effects on postnatal skeletal development of the offspring.

In pregnant rodents, fetal exposure to IH induces DNA damage in GABAergic nerve receptors in the cerebellum, neuronal apoptosis, and cardiovascular disorders such as hypertension and cardiac hypertrophy in the offspring (Chen et al., 2018). Gestational IH alters the response of the rat offspring to a subsequent postnatal inflammatory challenge in a gender-dependent manner (Johnson et al., 2018). It results in a significant reduction in endurance motor function in male adolescent offspring rats, associated with potential metabolic alterations and reduced capillary density in the diaphragm and anterior tibial muscles (Wongkitikamjorn et al., 2023). Furthermore, it induces mitochondrial impairment in the geniohyoid muscles of male offspring in the craniofacial region (Wongkitikamjorn et al., 2022). However, it is unknown whether gestational IH is a risk factor for craniofacial growth and development in offspring.

Children diagnosed with OSA have thinner cortical mandibular bones compared to healthy children. A negative correlation between OSA severity and mandibular cortical bone thickness was also found (Eimar et al., 2019). Children diagnosed with OSA by polysomnography have thinner cortical bone compared with children at low risk for OSA (Fernandes Fagundes et al., 2021), which suggests the interaction between mandibular bone development and pediatric OSA. Cortical bone thickness has a high accuracy in detecting osteoporosis (Calciolari et al., 2015). Furthermore, neonatal rats exposed to IH display underdeveloped mandibular ramus/condyles owing to the decreased mRNA level of SOX9 (*Sox9*), a key regulator for chondrogenesis in the mandible and limb (Lekvijittada et al., 2021). However, it is unclear whether maternal IH during pregnancy is a risk factor for the growth of mandible and long bone in offspring. This study aimed to clarify the effects of gestational IH on mandibular growth and development and to investigate cartilage metabolism-related marker expression in the mandibular condyle.

## 2 Materials and methods

### 2.1 Experimental model

Thirteen-week-old Sprague-Dawley pregnant rats were randomly exposed to normoxia as sham treatment (control group) and IH (IH group) (*n* = 8 each) at a rate of 20 cycles per hour (nadir, 4% oxygen; peak, 21% oxygen; 0% CO_2_), for eight hours/day during the 12-h “lights on” period, from gestation day (GD) 7–20, as previously described (Wongkitikamjorn et al., 2022; Wongkitikamjorn et al., 2023). All pups from both groups were born naturally under normoxia and maintained with their mothers until weaning. We randomly chose five or six of pups from each mother rat for this study, and pups were anesthetized with isoflurane and euthanized at 5 and 10 weeks of age. Nine 5-week-old male, nine 5-week-old female, nine 10-week-old male, and nine 10-week-old female pups were used in all analyses. All rats were allowed free access to food and water during the experimental period. At 5 and 10 weeks of age, male and female offspring were anesthetized with isoflurane and euthanized, and their bones were immediately fixed in 4% paraformaldehyde. The experimental procedures used in this study were approved by the Institutional Animal Care and Use Committee of the Tokyo Medical University (ethics approval number: H31-0011).

### 2.2 Three-dimensional micro-computed tomography analysis

The structure and bone mineral density (BMD) of the mandibular and tibial bones were analyzed using micro-computed tomography (micro-CT; inspeXio [SMX-100CT], Shimadzu, Kyoto, Japan) and three-dimensional image analysis software (TRI/3D-BON; RATOC System Engineering, Tokyo, Japan). The tibial length of the rat pups was measured as an index of systemic growth. Mandibular growth in rat pups was established and evaluated using five landmarks based on previous studies (Hong et al., 2021a; Hong et al., 2021b). Bone mineral density (BMD) of the mandibular and tibial condylar heads was measured as previously described (Hong et al., 2021a; Hong et al., 2021b).

### 2.3 Skeletal muscle analysis

To evaluate skeletal muscle growth, masseter, temporalis, digastric, and soleus muscles were collected from each rat litter. The weights of these muscles were measured on the right and left sides and the average was calculated.

### 2.4 Real-time polymerase chain reaction (RT-PCR) analysis

For RNA extraction, the left side of the mandibular condyle and tibial head were dissected under a stereomicroscope, frozen at −80°C, and homogenized. Total RNA was isolated from homogenized samples using a PureLink FFPE total RNA isolation kit (Invitrogen, Carlsbad, CA, United States), according to the manufacturer’s protocol. cDNA was synthesized from total RNA using a High-Capacity cDNA Reverse Transcription Kit (Applied Biosystems, Foster City, CA, United States). Real-time PCR was performed in triplicate for each sample using a 7500 Real-Time PCR System (Applied Biosystems). Polymerase chain reaction analyses were conducted using gene-specific primers and fluorescently labeled SYBR Green probes (Takara Bio, Shiga, Japan). Appropriate primers were chosen for real-time PCR amplification of *Sox9* (forward primer: 5′-aga​ggt​ttc​aaa​tgc​agt​gag​cta-3, reverse primer: 5′-cca​tga​cac​acg​ctt​gca​ga-3′), *Bmp2* (forward primer: 5′-tta​gac​gga​ctg​cgg​tct​cct​aa-3′, reverse primer: 5′-ggg​aag​cag​caa​cac​tag​aag​aca-3′), *Alp* (forward primer: 5′-act​gaa​ctg​ctg​gcc​ctt​gac-3; reverse primer: 5′-tca​ggt​tgt​tcc​gat​tca​act​cat​a-3), and *Hif1a* (forward primer: 5′-tct​agt​gaa​cag​gat​gga​atg​gag-3; reverse primer, 5-tcg​taa​ctg​gtc​agc​tgt​ggt​aa-3′). The thermocycling conditions used were 95°C for 30 s, followed by 40 cycles of 95°C for 5 s, and 60°C for 34 s. The threshold cycle (Ct) values of the target mRNAs (*Sox9*, *Bmp2*, *Alp*, and *Hif1a*) were normalized to the Ct values of the internal control (*GAPDH*) in the same sample (ΔCt = Ct_target_—Ct_GAPDH_), followed by normalization to the control (ΔΔCt = ΔCt_IHgroup_—ΔCt_Cgroup_). The fold-change in expression was calculated as the relative quantification value (RQ; 2^−ΔΔCT^) (Livak and Schmittgen, 2001).

### 2.5 Statistical analyses

Data are shown as the mean ± standard error (SE). The normality of the data was assessed using the Shapiro-Wilk test. The control and experimental groups were compared using an unpaired *t*-test, and statistical significance was accepted at a *p*-value < 0.05. All statistical analyses were performed using IBM SPSS Statistics Version 28.0.1 (Chicago, IL, United States).

## 3 Results

### 3.1 Systemic growth in rat offspring

**FIGURE 1:**
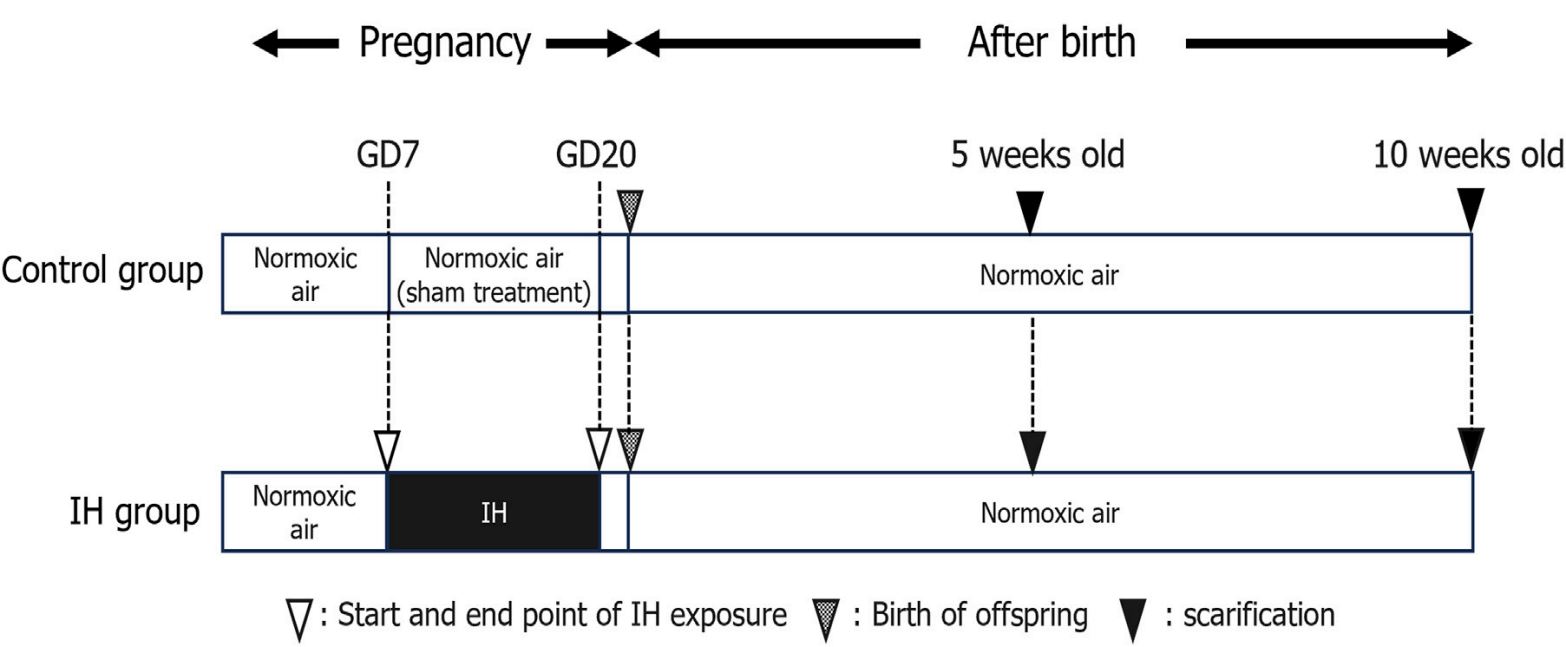
Experimental timeline. Abbreviations: GD, gestational day; IH, intermittent hypoxia, N, normoxic.

The effects of maternal sleep-disordered breathing (such as gestational OSA) on the birth of children are widely reported and fetal growth in women with OSA is slowed in the third trimester of pregnancy (Kneitel et al., 2018). Maternal snoring during pregnancy, a manifestation of sleep-disordered breathing, is a risk factor for adverse delivery outcomes such as cesarean section and small births for the number of weeks of gestation (Micheli et al., 2011; O’Brien et al., 2013). In addition, maternal snoring is implicated in the risk of hypertension and growth retardation in logistic regression analysis controlling for weight, age, and smoking (Franklin et al., 2000). In this experiment, pregnant rats were exposed to IH from GD7 to GD20, and delivery was completed on GD21 (Figure 1), and all pups from both groups were born naturally under normoxia and maintained with their mothers until weaning.

**FIGURE 2:**
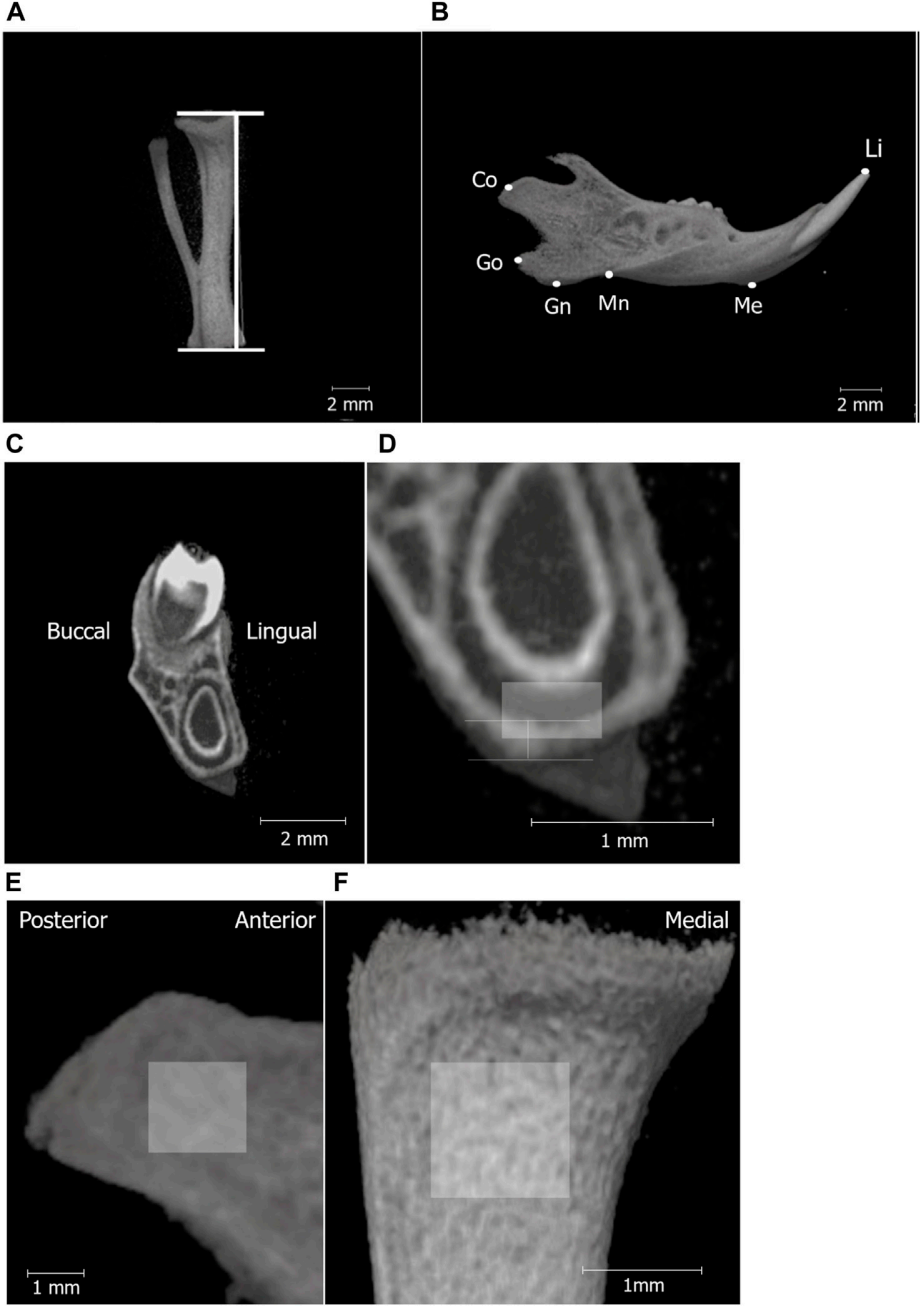
Micro-CT analysis landmarks for measuring the tibia and mandibular bones. Micro-CT images of the tibia **(A)**; line segment represents tibial length and separated hemi-right mandible **(B)**. The definitions of the landmarks and variables in the mandible are shown in Table 1. **(C)** shows the high-magnification micro-CT frontal image **(D)** of the mandible around the first molar (M1). The vertical line in **(D)** shows the area to measure cortical bone thickness below M1, and the box indicates the ROI for BMD measurement in the mandibular corpus (0.3 mm × 0.5 mm). Region of interest (ROI) for BMD measurements in the mandibular condyle (1.5 mm × 1.5 mm) **(E)**, tibia (1 mm × 1 mm) **(F)**.

**FIGURE 3:**
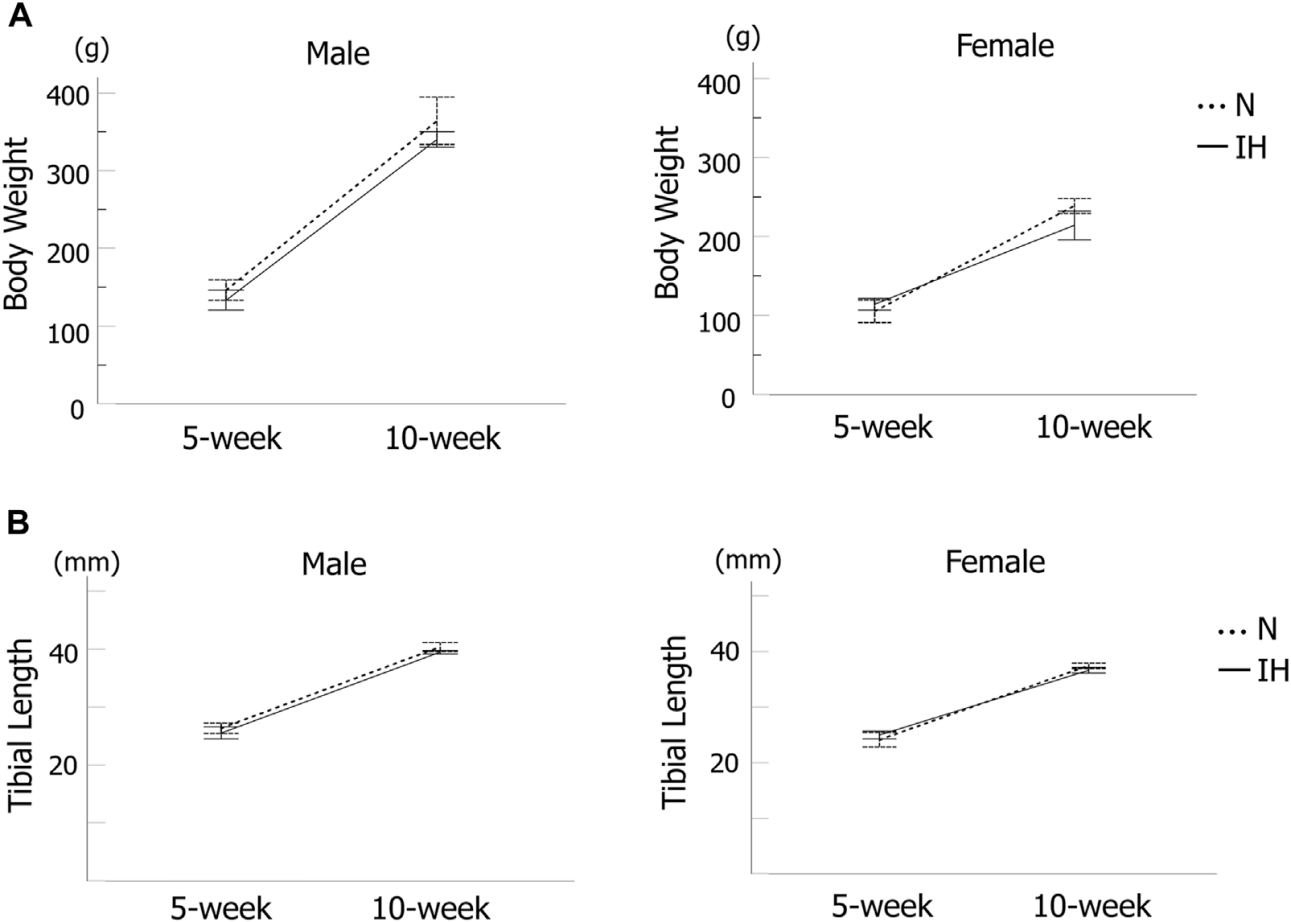
Systemic growth of offspring rats. **(A)** Body weight changes in male and female offspring at 5 and 10 weeks after birth. **(B)** Tibia length of male and female offspring at 5 and 10 weeks after birth. Data are presented as the mean ± SE for each group. *p < 0.05.

Body weight and tibial length (Figure 2A) of the rat offspring were measured as an index of whole-body growth. The body weight of all offspring rats gradually increased, with no significant difference between the control and IH groups at 5 and 10 weeks after birth (Figure 3A). Moreover, there was no significant difference in tibial length between the control and IH groups (Figure 3B).

### 3.2 Effects of gestational IH on mandibular condylar growth and cortical bone structure in rat offspring

Mandibular growth in rat pups was evaluated using five landmarks (Figure 2B; Table 1).

**TABLE 1:** Definitions of landmarks and variables in the mandible.

Landmarks	
Co	The most posterior and superior points on the mandibular condyle
Go	The most posterior point on the mandibular ramus
Gn	The most inferior point on the ramus that lies on a perpendicular bisector of the Go-Mn line
Me	The most inferior and anterior points on the lower border of the mandible
Mn	The most concave portion of the inferior border of the mandibular corpus
Li	The most anterior and superior points on the alveolar bone of the mandibular incisor
Linear measurements	
Co-Li	Total mandibular length
Co-Me	Length from the condylar head to Me
Co-Go	Length from the condylar head to Go
Co-Gn	Ramus height

**FIGURE 4:**
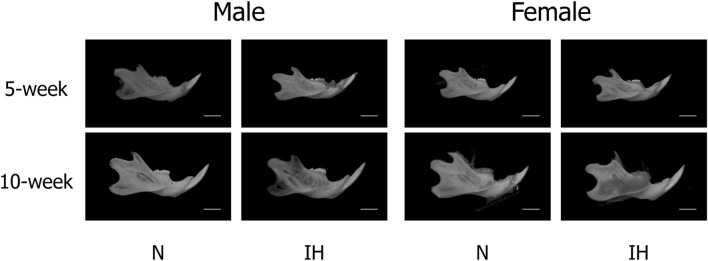
CT-images of mandibular bone. Representative CT images of male and female offspring mandibles in the normoxic (N) and gestational IH (IH) groups at 5 weeks of age (5-week) and 10 weeks (10-week) of age.

**FIGURE 5:**
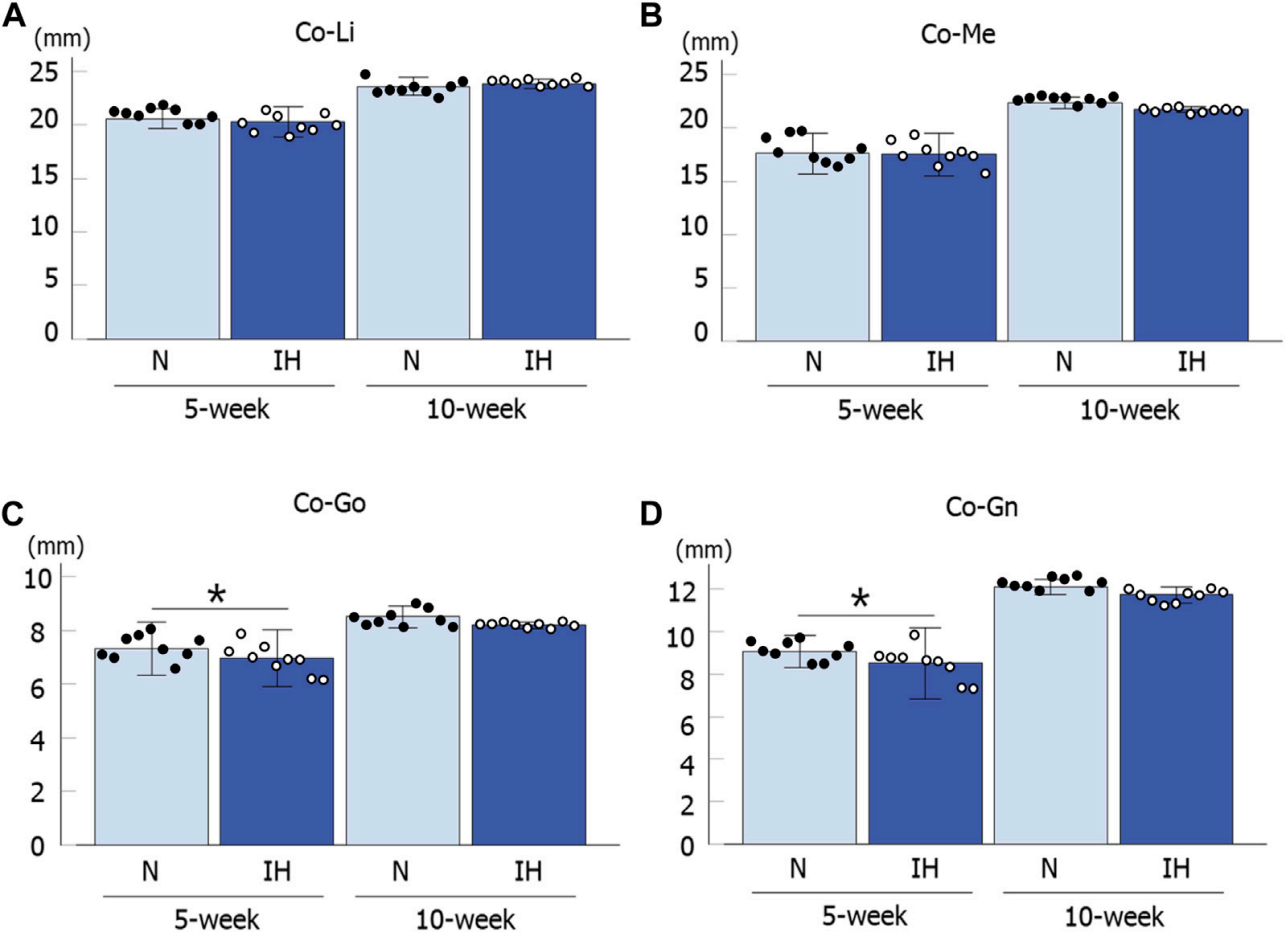
Mandibular linear measurements in male offspring rats. Comparison of changes in mandibular growth between normoxic (N) and IH male offspring at 5 and 10 weeks of age **(A–D)**. Data are presented as the mean ± SE for each group. *p < 0.05.

Among the four parameters in the mandible of male offspring, Co-Go [6.96 ± 0.18 mm in the IH group vs. 7.33 ± 0.16 mm in the N group, *p* = 0.041] and Co-Gn [8.52 ± 0.28 mm in the IH group vs. 9.06 ± 0.12 mm in the N group, *p* = 0.041] parameters related to the condylar length were significantly shorter in the gestational IH offspring than in the control offspring at 5 weeks of age (Figures 4, 5A–D). Meanwhile, there was no significant difference in Co-Go and Co-Gn between the gestational IH offspring and the control offspring at 10 weeks of age.

**FIGURE 6:**
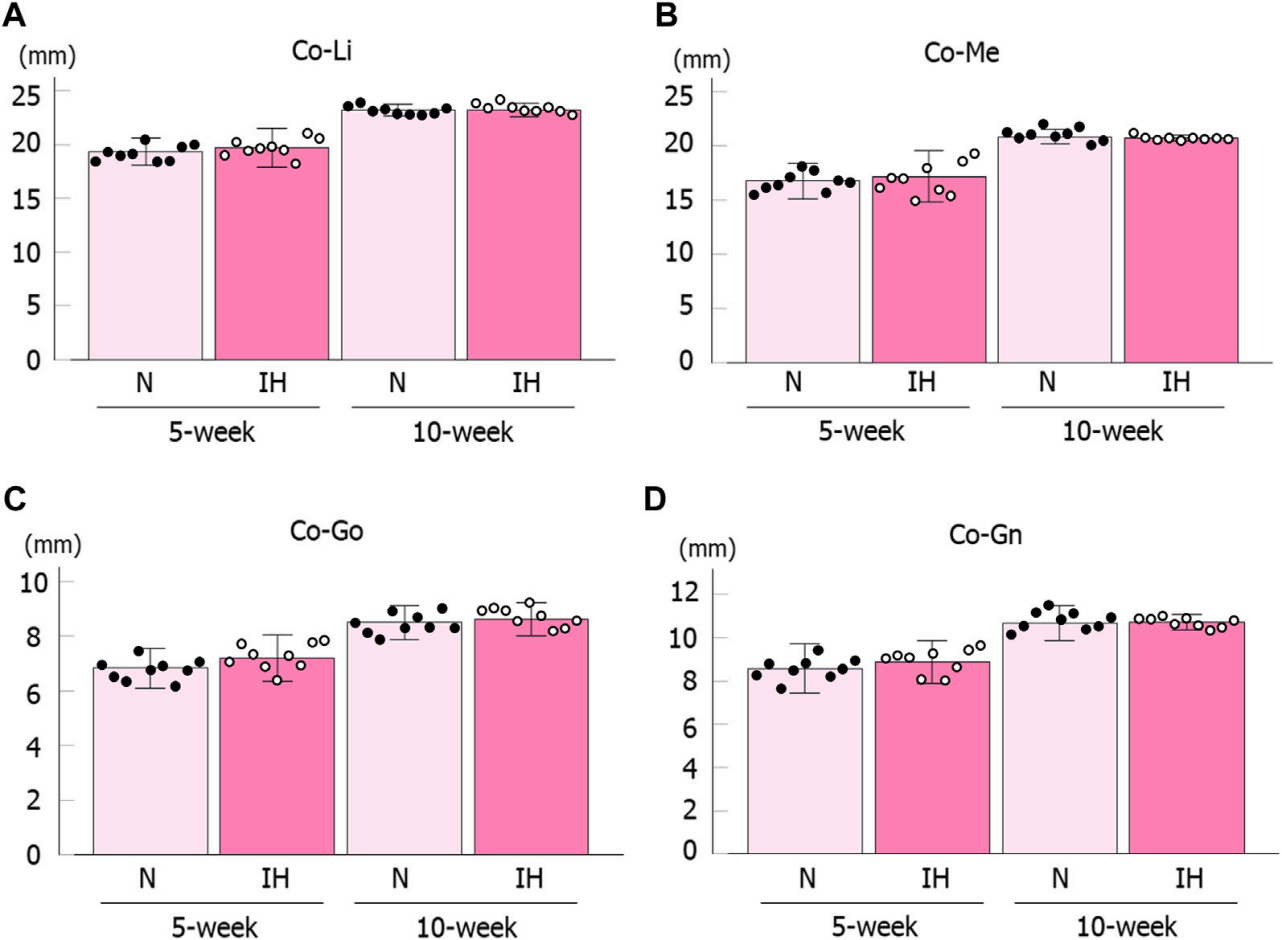
Mandibular linear measurements in female offspring rats. Comparison of changes in mandibular growth between normoxic (N) and IH female offspring at 5 and 10 weeks of age **(A–D)**. Data are presented as the mean ± SE for each group. *p < 0.05.

In contrast, linear measurements in the mandible were comparable between the control and IH groups in female offspring at 5 and 10 weeks of age (Figures 4, 6A–D).

The cortical bone thickness at the inferior edge of the mandible was measured at the mandibular first molar region (Figures 2C, D).

**FIGURE 7:**
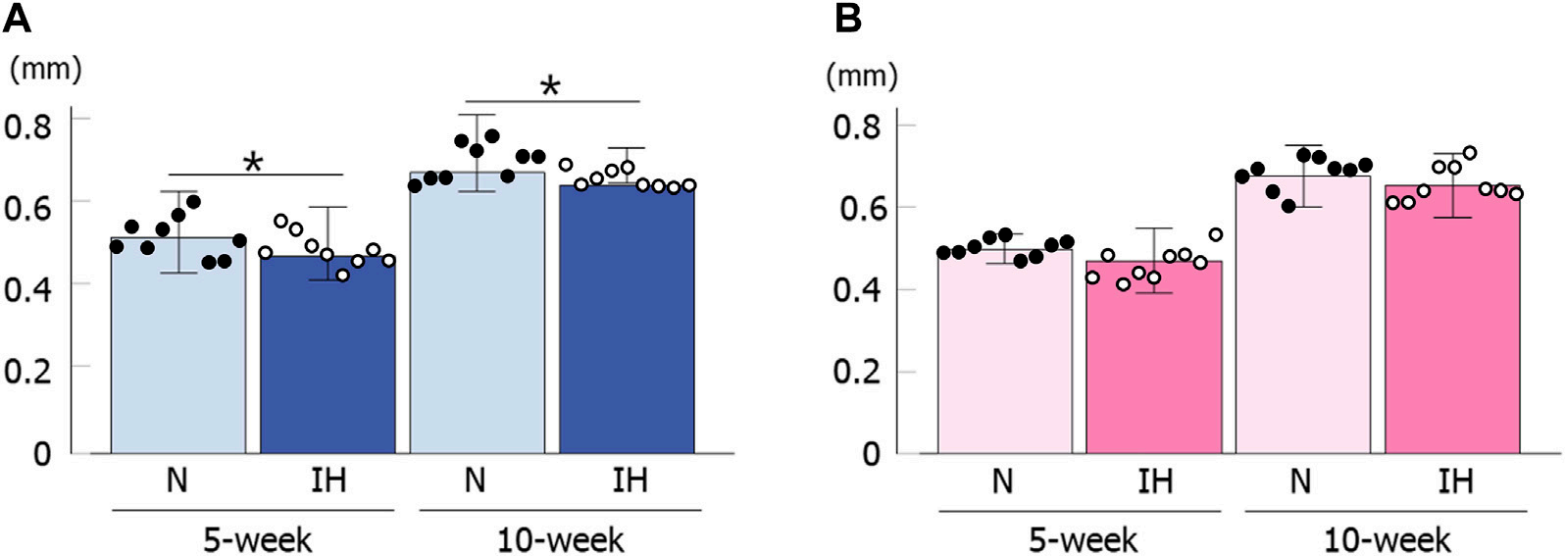
Cortical bone thickness below the mandibular M1. Comparison of changes in male **(A)** and female **(B)** offspring at 5 and 10 weeks after birth. Data are presented as the mean ± SE for each group. *p < 0.05.

Cortical bone thickness at the inferior edge of the mandible was significantly thinner in the gestational IH male offspring than in the control male offspring at 5 and 10 weeks of age [5 weeks male rat: 0.48 ± 0.15 mm in the IH group vs. 0.52 ± 0.16 mm in the N group, *p* = 0.029; 10 weeks male rat: 0.65 ± 0.011 mm in the IH group vs. 0.68 ± 0.018 mm in the N group, *p* = 0.040] (Figure 7A). In contrast, there was no significant difference in cortical bone thickness around the mandibular M1 at 5 and 10 weeks of age in female offspring (Figure 7B).

### 3.3 Bone mineral density in the mandible and tibia

Bone mineral density (BMD) of the mandibular and tibial condylar heads was measured as previously described (Figures 2E, F).

**FIGURE 8:**
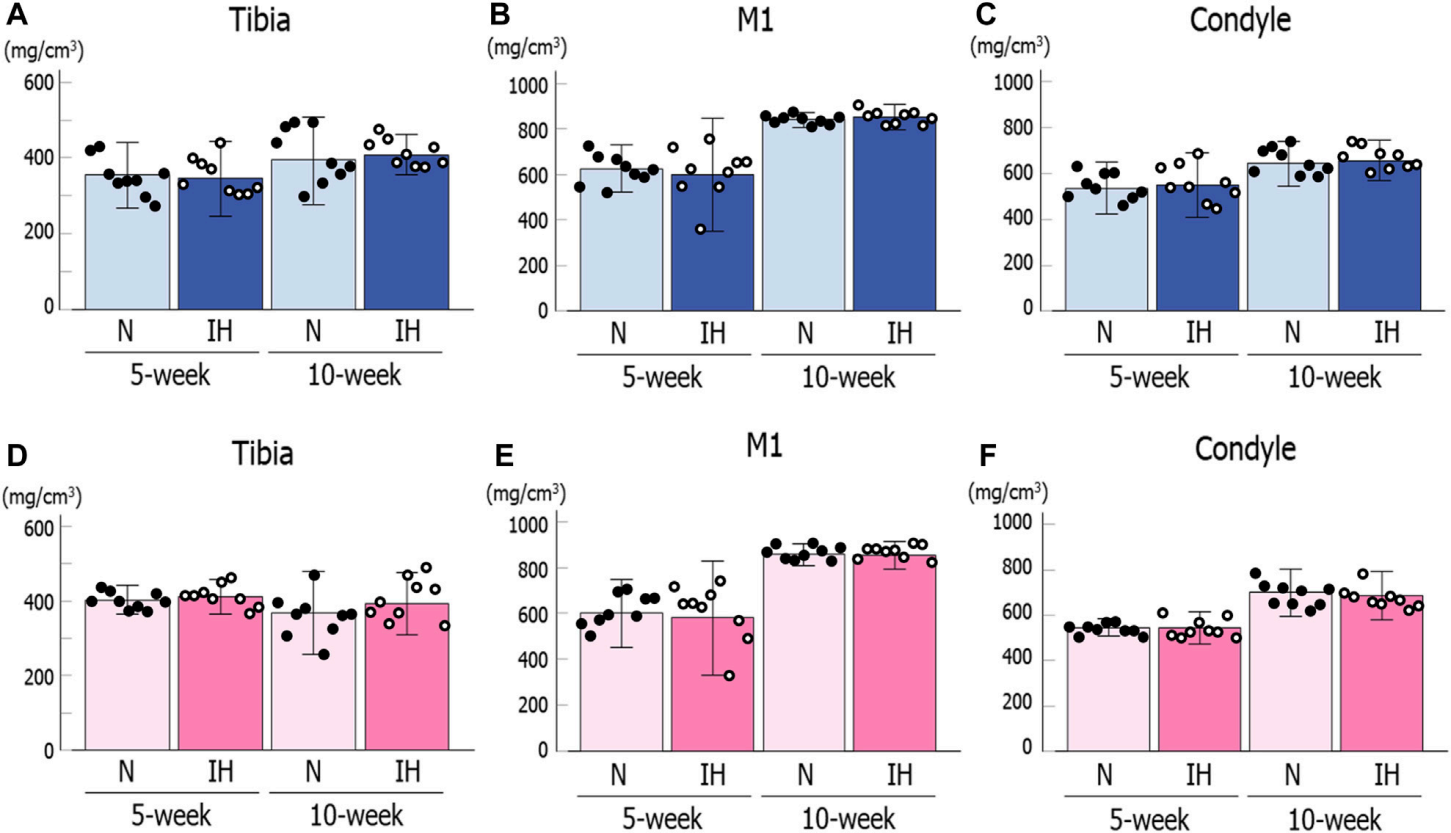
Bone mineral density (BMD) for the mandible and tibia of male and female offspring rats. Mandibular condylar head **(A)**, mandibular corpus below M1 **(B)**, and tibial head **(C)** of male offspring. Mandibular condylar head **(D)**, mandibular corpus below M1 **(E)**, and tibial head **(F)** of female offspring. Data are presented as the mean ± SE for each group. *p < 0.05.

BMD in the mandibular condyle and mandibular corpus below M1 showed no significant difference between the control and IH male offspring at 5 and 10 weeks of age based on micro-CT analysis (Figures 8A, B). Moreover, BMD in the tibial head was comparable between both groups of male offspring (Figure 8C). The female offspring also showed comparable values between the control and IH groups at 5 and 10 weeks of age in the mandibular condyle, mandibular corpus below M1, and tibial head (Figures 8D–F).

### 3.4 Skeletal muscle weight analysis

We measured the weight of masticatory muscles to explore the possibility of undergrowth of masticatory muscle in IH offspring since mandibular formation in young rats is potently affected by masticatory and chewing functions (Bresin et al., 1999; Kün-Darbois et al., 2015). There were no significant differences in the weight of the masseter, temporalis, gastric, and soleus muscles between the control and IH male offspring at 5 and 10 weeks of age (Supplementary Figure S1). However, there was a significant difference in skeletal muscle weight of the masseter, temporalis, and digastric muscles between the control and IH female offspring (Supplementary Figure S2).

### 3.5 Differential gene expression of SOX9 and HIF-1α in the mandibular condyle of gestational IH offspring

The mandibular condylar cartilage is the center of greatest growth in the mandible (Copray et a1., 1988). SOX9 triggers chondrogenic differentiation via TGF-β/Smad signaling (Ikeda et al., 2005; Furumatsu et al., 2013). Alkaline phosphatase (ALP) and bone morphogenetic protein-2 (BMP-2) induce osteogenesis and osteogenic transformation (Kuru et al., 1999; Selvig et al., 2002). Thus, we checked the mRNA levels of SOX9, BMP2, and ALP in the underdeveloped mandibular condyles of IH offspring.

**FIGURE 9:**
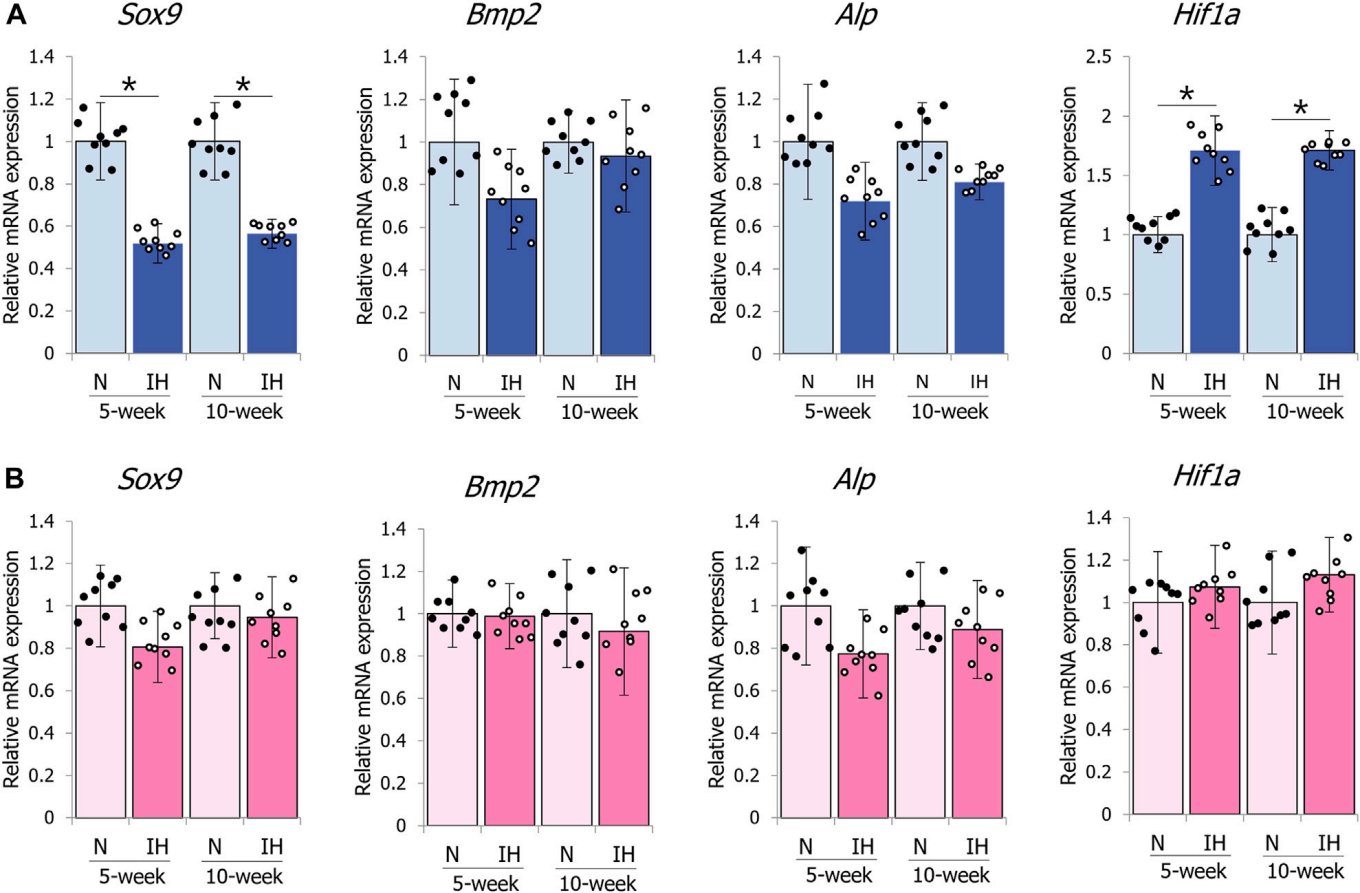
mRNA expression levels of genes associated with chondrocyte and bone metabolism in mandibular condyle. Relative mRNA expression levels of SOX9 (*Sox9*), BMP2 (*Bmp2*), ALP (*Alp*), and HIF-1α (*Hif1a*) in 5- and 10-week male **(A)** offspring, 5- and 10-week female **(B)** offspring. Data are presented as the mean ± SE for each group. *p < 0.05.

Gestational IH significantly decreased the mRNA level of the chondrogenesis marker SOX9 (*Sox9*) and increased the mRNA level of the hypoxia-inducible factor HIF-1α (*Hif1a*) in the mandibular condylar cartilage of the 5- and 10-week-old male offspring (Figure 9A). However, the mRNA levels of SOX9, HIF-1α, BMP2 (*Bmp2*), and ALP (*Alp*) in the mandibular condyle of the female offspring were comparable between both groups (Figure 9B).

**FIGURE 10:**
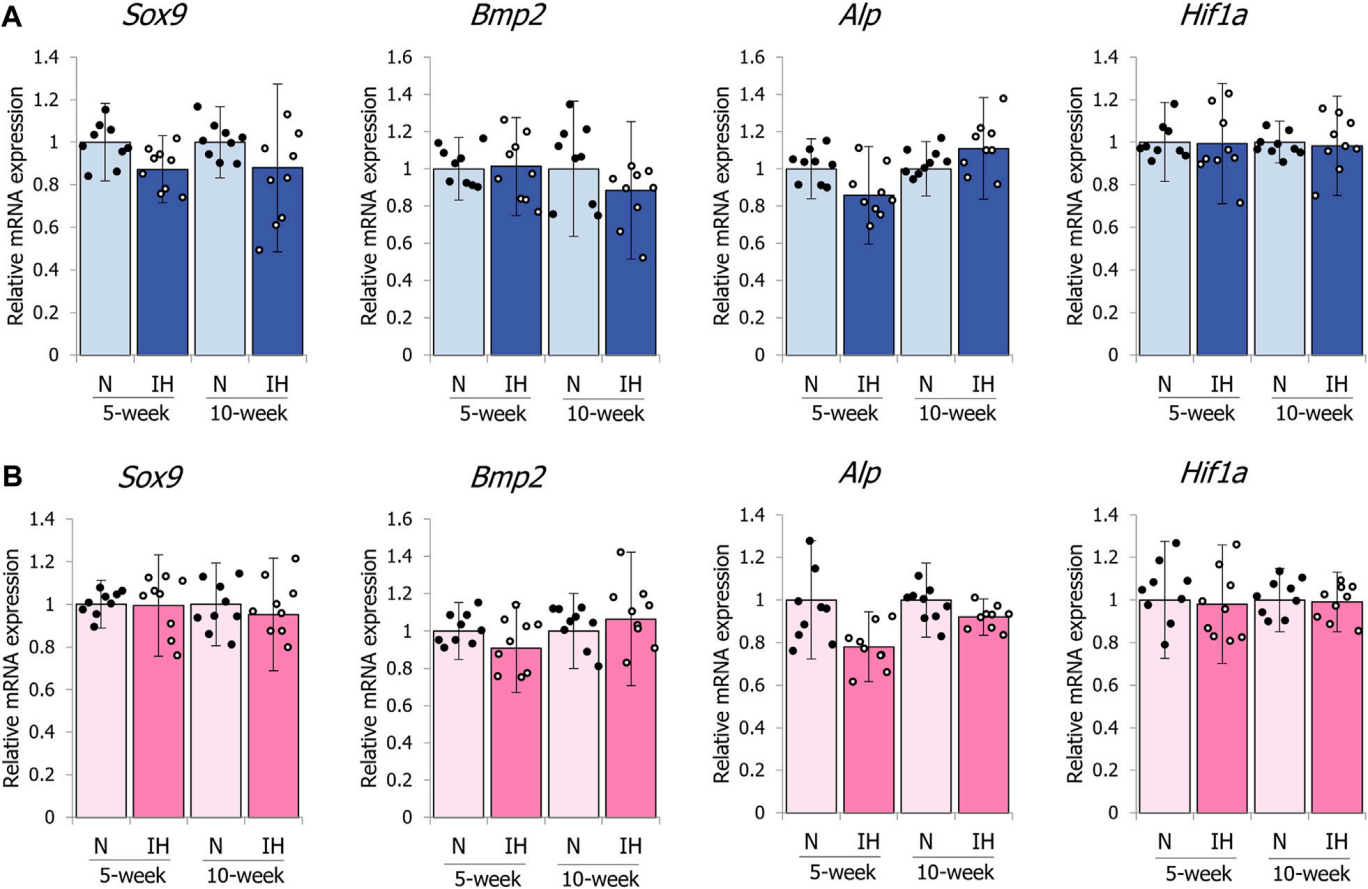
mRNA expression levels of genes associated with chondrocyte and bone metabolism in the tibia. Relative mRNA expression levels of SOX9 (*Sox9*), BMP2 (*Bmp2*), ALP (*Alp*), and HIF-1α (*Hif1a*) in 5- and 10-week female **(A)** offspring, 5- and 10-week female **(B)** offspring. Data are presented as the mean ± SE for each group. *p < 0.05.

In contrast to the mandibular condylar cartilage, gestational IH did not affect the expression levels of these genes in the tibial cartilage of male and female offspring (Figures 10A, B).

## 4 Discussion

This is the first study demonstrating gender-dependent modification of mandibular growth with reduced SOX9 (*Sox9*) mRNA expression and increased HIF1-α (*Hif1a*) mRNA expression in the condylar cartilage in male offspring exposed to gestational IH. A 2-week IH exposure during prenatal development caused underdeveloped mandibular ramus/condyle growth revealed in the Co-Go and Co-Gn directions in male offspring, but not in female offspring. IH exposure during prenatal development also caused growth failure of the cortical bone below M1 in male offspring. Although mandibular formation in young rats is potently affected by masticatory and chewing functions (Grünheid et al., 2011; Inoue et al., 2019), the weight of the masticatory muscles was comparable in male offspring of the IH and control groups (Supplementary Figure S1). Our findings indicate the possibility of gender-dependent detrimental consequences on cartilaginous craniofacial development in offspring exposed to gestational IH, a common symptom of OSA during pregnancy.

Gestational IH reduces type IIA fiber size in the geniohyoid muscle of the offspring rat from the gestational OSA model (Wongkitikamjorn et al., 2022). They suggest that mitochondrial metabolism is impaired owing to gestational IH and changes in oxidative myofibers in the geniohyoid muscles, which may be attributable to the sensitivity of mitochondria of the geniohyoid muscle to gestational IH. Moreover, exposure to gestational IH decreases running endurance in the adolescent pups (Wongkitikamjorn et al., 2023). The expression of genes related to glucose and lipid metabolism and the protein levels of phosphorylated AMPK and AKT decreased in pregnant IH rats. Furthermore, the gene expression of adiponectin receptors 1 and 2 significantly decreased in the respiratory (diaphragm) and limb (tibialis anterior) muscles (Wongkitikamjorn et al., 2023). These results suggest that the respiratory and limb muscles are vulnerable to IH during pregnancy. Chen et al. (2018) showed that maternal exposure to IH reduced body weight and tibial length in male and female offspring, with male rats more prone to hypertension and left ventricular dysfunction. On the other hand, Song et al. (2022) showed that pups born to gestational IH exhibited catch-up growth and were comparable to the controls by 5 weeks of age although they were significantly smaller at birth. In this study, we observed no significant difference in the body weight and tibial length of newborn rats exposed to prenatal IH at 5 and 10 weeks of age.

In rat fetuses, the mandibular condyle is recognized as mesenchymal condensation at GD14.5, and the glenoid fossa of the mandible is recognized at GD15.5 (Yamaki et al., 2005). At the fetal mandibular condyle, differentiation of mesenchymal condensation into chondrocytes is initiated by GD16.5. At GD17, the secondary cartilage of the mandibular condylar process arises slightly dorsal to Meckel’s cartilage. At GD18, the secondary cartilage becomes relatively larger and starts forming a cudgel-like structure of the mandibular condylar process (Tomo et al., 1997). Endochondral ossification was observed at GD17.5. Meanwhile, part of the molar socket region already appears at GD17. In this study, the IH period from GD7 to GD20 covered the period of early embryonic development of the condyle and molar socket regions of the rat mandible. Furthermore, rat embryos generally become more sensitive to hypoxia during mid-gestation, GD13 to GD16 in the uterine clamping model (Ritchie et al., 2017). Thus, the susceptibility of the rat mandible to gestational IH may depend on the fetal growth stage.

A study conducted in children found that children with OSA had a smaller mandibular cortical bone width than children at low risk for OSA (Fernandes Fagundes et al., 2021). Pediatric OSA is associated with craniofacial vertical growth retardation and malocclusion, which are related to mandibular cortical bone thickness. Young rats raised under the IH condition also exhibit the craniofacial changes such as narrowing in the lower dental arch (Hosomichi et al., 2017) and the reduced mandibular growth (Oishi et al., 2016a; Oishi et al., 2016b). Badran et al. (2019) suggested that IH during pregnancy can induce placental hypoxia and oxidative stress, exposing the fetus to IH and affect mandibular growth. Rats born from the pregnant IH group in this study showed a decrease in cortical bone thickness below M1. Our pregnant models were exposed to IH on days 7–20 of pregnancy-induced placental hypoxia and oxidative stress. Exposure to hypoxia reduces rat osteoblast proliferation and inhibits bone formation *in vitro* (Utting et al., 2006). Our results are in accord with a decrease in cortical bone width in the mandible in children with sleep-disordered breathing (Eimar et al., 2019).

The growth of the mandible in rats was at its maximum from 4 weeks to 8 weeks after birth, and continued to grow until the 16th week (Kim et al., 2018). Meanwhile, the cortical bone of the mandible rapidly reaches almost complete mineralization in rats compared to humans, which suggests that cortical bone remodeling change little in adolescent rats (Matsumoto et al., 2011; Lad, 2023). In our study, the morphological growth (cartilage growth) of the mandible in the male IH group returned to that of the control group (Figures 5C, D), but not in the cortical bone (Figure 7A). This may be owing to the difference in the timing of cartilage and bone growth, and the fact that rat cortical bone does not undergo remodeling. In addition, our data suggest that some catch-up of skeletal growth may occur during the adolescent stage in the offspring, although gestational IH has a potent effect on skeletal growth during the early growth stage.

Hypoxia during pregnancy causes differences in the development of the heart, kidneys, and central nervous system between the sexes and it affects males but not females (Bourque et al., 2013; Thompson et al., 2020; Tong et al., 2023; Wilson et al., 2023). In our study, we found sex differences in the growth and development of mandibular and cortical bone owing to exposure to IH during pregnancy. There are no reports on osteochondral development except for our study, and it is possible that hypoxia during pregnancy may have caused gender differences in osteochondral development, as in other organs. However, the detailed mechanism behind the gender difference in this study is unclear and further research is warranted.

Quantitative RT-PCR analysis of the mandibular epiphyseal cartilage of male offspring from pregnant IH mothers revealed upregulated HIF-1α (*Hif1a*) and downregulated SOX9 (*Sox9*) mRNA levels. There is increased HIF-1α expression in the brains of offspring rodents exposed to prenatal hypoxia and maternal smoking (Chan et al., 2016; Wang et al., 2021; Wilson et al., 2022), which suggests that gestational hypoxia increases oxidative stress in young adult offspring. Furthermore, hypoxia and osteogenesis are related via activation of the HIF pathway, which causes significant changes in bone growth when oxygen levels are low (Liu and Simon, 2004). HIF-1α regulation of SOX9 is necessary to maintain the differentiation of hypoxic prechondrogenic cells during skeletal growth (Amarilio et al., 2007), and HIF-1α expression levels correlate with SOX9 levels. However, we observed reduced SOX9 mRNA levels in the epiphyseal cartilage mandible of male IH offspring, despite increased HIF-1α mRNA levels. A previous histological study in neonatal IH rats (Hong et al., 2021a) demonstrates that IH shifts proliferation and maturation in the mandibular condyle fibrocartilage toward hypertrophic differentiation and ossification by downregulating mRNA levels of SOX9 and TGF-β in male rats, which showed mandibular growth restriction. SOX9 can suppress chondrocyte maturation and the osteoblast phenotype from the proliferative to early hypertrophic and developmental stages (Dy et al., 2012), which may provide a possible explanation for the mandibular growth retardation and hypertrophic differentiation of mandibular cartilaginous chondrocytes caused by the decreased SOX9 levels in gestational IH offspring.

In this study, we found sex differences in mandibular bone growth and mRNA expression of SOX9 and HIF-1α in the adolescent offspring of pregnant rats exposed to IH. A previous study in a rat gestational IH model focused on the different negative effects between male and female offspring (Wilson et al., 2022), which indicated that exposure to IH during pregnancy can mediate the developmental programming of both cortical and subcortical pathways, resulting in long-term negative consequences for male offspring compared with female offspring. Pae et al., 2011; Pae and Harper, 2023) revealed that IH exposure for 1 h immediately after birth causes higher nor-epinephrine levels in the blood and disturbance of mandibular bone remodeling for at least the first five postnatal weeks in male neonatal rats, in contrast to female pups. They also suggested that both bone deficiencies and potential metabolic alterations are sex-specific. β2-adrenergic receptors, which are targets of norepinephrine, are predominantly expressed in bone cells, and skeletal growth is potently regulated by sympathetic neurotransmission. Hong et al. (2021a) showed that postnatal IH resulted in higher leptin levels and underdeveloped mandibular ramus/condyles in adolescent male IH rats. In addition, reduced bone growth of the mandible with the activation of β2-adrenergic receptors is restored by intraperitoneal administration of a β2-adrenergic antagonist, which changes RANKL expression in the growing condyle (Hong et al., 2021b). Although further investigations are necessary to clarify both the serum levels of nor-epinephrine and the neuroskeletal regulatory pathway after gestational IH, one possible explanation may be that there is an impaired neuroskeletal regulatory pathway in the male offspring after gestational IH.

A limitation of this study was the absence of epigenomic data to show the possible SOX9-directed pathways under gestational IH in offspring rats. Moreover, we need to examine details of the gender-dependent pathway of impaired craniofacial bone growth in offspring to clarify why male offspring were more susceptible to gestational IH than female offspring.

## Data Availability

The original contributions presented in the study are included in the article/Supplementary Material, further inquiries can be directed to the corresponding author.

## References

[B1] Amarilio R. Viukov S. V. Sharir A. Eshkar-Oren I. Johnson R. S. Zelzer E. (2007). HIF1alpha regulation of Sox9 is necessary to maintain differentiation of hypoxic prechondrogenic cells during early skeletogenesis. Development 134 (21), 3917–3928. 10.1242/dev.008441 17913788

[B2] Badran M. Abuyassin B. Ayas N. Laher I. (2019). Intermittent hypoxia impairs uterine artery function in pregnant mice. J. Physiol. 597 (10), 2639–2650. 10.1113/JP277775 31002746 PMC6826231

[B3] Bourque S. L. Gragasin F. S. Quon A. L. Mansour Y. Morton J. S. Davidge S. T. (2013). Prenatal hypoxia causes long-term alterations in vascular endothelin-1 function in aged male, but not female, offspring. Hypertension 62 (4), 753–758. 10.1161/HYPERTENSIONAHA.113.01516 23940196

[B4] Bresin A. Kiliaridis S. Strid K. G. (1999). Effect of masticatory function on the internal bone structure in the mandible of the growing rat. Eur. J. Oral. Sci. 107 (1), 35–44. 10.1046/j.0909-8836.1999.eos107107.x 10102749

[B5] Calciolari E. Donos N. Park J. C. Petrie A. Mardas N. (2015). Panoramic measures for oral bone mass in detecting osteoporosis: a systematic review and meta-analysis. J. Dent. Res. 94 (3), 17S–27S. 10.1177/0022034514554949 25365969 PMC4541087

[B6] Chan Y. L. Saad S. Pollock C. Oliver B. Al-Odat I. Zaky A. A. (2016). Impact of maternal cigarette smoke exposure on brain inflammation and oxidative stress in male mice offspring. Sci. Rep. 6, 25881. 10.1038/srep25881 27169932 PMC4864383

[B7] Chen L. Zadi Z. H. Zhang J. Scharf S. M. Pae E. K. (2018). Intermittent hypoxia *in utero* damages postnatal growth and cardiovascular function in rats. J. Appl. Physiol. 124 (4), 821–830. 10.1152/japplphysiol.01066.2016 29357521

[B8] Copray J. C. Dibbets J. M. Kantomaa T. (1988). The role of condylar cartilage in the development of the temporomandibular joint. Angle Orthod. 58 (4), 369–380. 10.1043/0003-3219(1988)058<0369:TROCCI>2.0.CO;2 3061315

[B9] Dy P. Wang W. Bhattaram P. Wang Q. Wang L. Ballock R. T. (2012). Sox9 directs hypertrophic maturation and blocks osteoblast differentiation of growth plate chondrocytes. Dev. Cell. 22 (3), 597–609. 10.1016/j.devcel.2011.12.024 22421045 PMC3306603

[B10] Eimar H. Al-Saleh M. A. Q. Cortes A. R. G. Gozal D. Graf D. Flores-Mir C. (2019). Sleep-disordered breathing is associated with reduced mandibular cortical width in children. JDR Clin. Trans. Res. 4 (1), 58–67. 10.1177/2380084418776906 30931759

[B11] Fernandes Fagundes N. C. d'Apuzzo F. Perillo L. Puigdollers A. Gozal D. Graf D. (2021). Potential impact of pediatric obstructive sleep apnea on mandibular cortical width dimensions. J. Clin. Sleep. Med. 17 (8), 1627–1634. 10.5664/jcsm.9262 33745506 PMC8656917

[B12] Franklin K. A. Holmgren P. A. Jönsson F. Poromaa N. Stenlund H. Svanborg E. (2000). Snoring, pregnancy-induced hypertension, and growth retardation of the fetus. Chest 117 (1), 137–141. 10.1378/chest.117.1.137 10631211

[B13] Fung A. M. Wilson D. L. Lappas M. Howard M. Barnes M. O'Donoghue F. (2013). Effects of maternal obstructive sleep apnoea on fetal growth: a prospective cohort study. PLoS One 8 (7), e68057. 10.1371/journal.pone.0068057 23894293 PMC3722214

[B14] Furumatsu T. Matsumoto E. Kanazawa T. Fujii M. Lu Z. Kajiki R. (2013). Tensile strain increases expression of CCN2 and COL2A1 by activating TGF-β-Smad2/3 pathway in chondrocytic cells. J. Biomech. 46 (9), 1508–1515. 10.1016/j.jbiomech.2013.03.028 23631855

[B15] Grünheid T. Langenbach G. E. Brugman P. Everts V. Zentner A. (2011). The masticatory system under varying functional load. Part 2: effect of reduced masticatory load on the degree and distribution of mineralization in the rabbit mandible. Eur. J. Orthod. 33 (4), 365–371. 10.1093/ejo/cjq084 20923936

[B16] Heindel J. J. Balbus J. Birnbaum L. Brune-Drisse M. N. Grandjean P. Gray K. (2015). Developmental origins of health and disease: integrating environmental influences. Endocrinology 156 (10), 3416–3421. 10.1210/EN.2015-1394 26241070 PMC4588819

[B17] Hong H. Hosomichi J. Maeda H. Ishida Y. Usumi-Fujita R. Yoshida K. I. (2021b). Selective β2-adrenoceptor blockade rescues mandibular growth retardation in adolescent rats exposed to chronic intermittent hypoxia. Front. Physiol. 12, 676270. 10.3389/fphys.2021.676270 34220541 PMC8247478

[B18] Hong H. Hosomichi J. Maeda H. Lekvijittada K. Oishi S. Ishida Y. (2021a). Intermittent hypoxia retards mandibular growth and alters RANKL expression in adolescent and juvenile rats. Eur. J. Orthod. 43 (1), 94–103. 10.1093/ejo/cjaa020 32219305

[B19] Hosomichi J. Kuma Y. I. Oishi S. Nagai H. Maeda H. Usumi-Fujita R. (2017). Intermittent hypoxia causes mandibular growth retardation and macroglossia in growing rats. Am. J. Orthod. Dentofac. Orthop. 151 (2), 363–371. 10.1016/j.ajodo.2016.02.033 28153167

[B20] Ikeda T. Kawaguchi H. Kamekura S. Ogata N. Mori Y. Nakamura K. (2005). Distinct roles of Sox5, Sox6, and Sox9 in different stages of chondrogenic differentiation. J. Bone Min. Metab. 23 (5), 337–340. 10.1007/s00774-005-0610-y 16133682

[B21] Inoue M. Ono T. Kameo Y. Sasaki F. Ono T. Adachi T. (2019). Forceful mastication activates osteocytes and builds a stout jawbone. Sci. Rep. 9 (1), 4404. 10.1038/s41598-019-40463-3 30890758 PMC6424982

[B22] Johnson S. M. Randhawa K. S. Epstein J. J. Gustafson E. Hocker A. D. Huxtable A. G. (2018). Gestational intermittent hypoxia increases susceptibility to neuroinflammation and alters respiratory motor control in neonatal rats. Respir. Physiol. Neurobiol. 256, 128–142. 10.1016/j.resp.2017.11.007 29174411 PMC5963968

[B23] Kim H. J. Park K. M. Tak H. J. Choi J. W. Kang S. H. Park W. (2018). Three-dimensional growth pattern of the rat mandible revealed by periodic live micro-computed tomography. Arch. Oral Biol. 87, 94–101. 10.1016/j.archoralbio.2017.12.012 29275154

[B24] Kneitel A. W. Treadwell M. C. O'Brien L. M. (2018). Effects of maternal obstructive sleep apnea on fetal growth: a case-control study. J. Perinatol. 38 (8), 982–988. 10.1038/s41372-018-0127-6 29785058 PMC6092194

[B25] Kün-Darbois J. D. Libouban H. Chappard D. (2015). Botulinum toxin in masticatory muscles of the adult rat induces bone loss at the condyle and alveolar regions of the mandible associated with a bone proliferation at a muscle enthesis. Bone 77, 75–82. 10.1016/j.bone.2015.03.023 25857689

[B26] Kuru L. Griffiths G. S. Petrie A. Olsen I. (1999). Alkaline phosphatase activity is upregulated in regenerating human periodontal cells. J. Periodontal Res. 34 (2), 123–127. 10.1111/j.1600-0765.1999.tb02231.x 10207841

[B27] Lad S. E. (2023). Absence of secondary osteons in femora of aged rats: implications of lifespan on Haversian remodeling in mammals. J. Morphol. 284 (7), e21600. 10.1002/jmor.21600 37313764

[B28] Lekvijittada K. Hosomichi J. Maeda H. Hong H. Changsiripun C. Kuma Y. I. (2021). Intermittent hypoxia inhibits mandibular cartilage growth with reduced TGF-β and SOX9 expressions in neonatal rats. Sci. Rep. 11 (1), 1140. 10.1038/s41598-020-80303-3 33441835 PMC7806651

[B29] Liu L. Simon M. C. (2004). Regulation of transcription and translation by hypoxia. Cancer Biol. Ther. 3 (6), 492–497. 10.4161/cbt.3.6.1010 15254394

[B30] Livak K. J. Schmittgen T. D. (2001). Analysis of relative gene expression data using real-time quantitative PCR and the 2(-Delta Delta C(T)) Method. Methods 25 (4), 402–408. 10.1006/meth.2001.1262 11846609

[B31] Matsumoto T. Ando N. Tomii T. Uesugi K. (2011). Three-dimensional cortical bone microstructure in a rat model of hypoxia-induced growth retardation. Calcif. Tissue Int. 88 (1), 54–62. 10.1007/s00223-010-9415-7 20848090

[B32] Micheli K. Komninos I. Bagkeris E. Roumeliotaki T. Koutis A. Kogevinas M. (2011). Sleep patterns in late pregnancy and risk of preterm birth and fetal growth restriction. Epidemiology 22 (5), 738–744. 10.1097/EDE.0b013e31822546fd 21734587

[B33] Nalivaeva N. N. Turner A. J. Zhuravin I. A. (2018). Role of prenatal hypoxia in brain development, cognitive functions, and neurodegeneration. Front. Neurosci. 12, 825. 10.3389/fnins.2018.00825 30510498 PMC6254649

[B34] O'Brien L. M. Bullough A. S. Owusu J. T. Tremblay K. A. Brincat C. A. Chames M. C. (2013). Snoring during pregnancy and delivery outcomes: a cohort study. Sleep 36 (11), 1625–1632. 10.5665/sleep.3112 24179294 PMC3792378

[B35] Oishi S. Shimizu Y. Hosomichi J. Kuma Y. Maeda H. Nagai H. (2016b). Intermittent hypoxia influences alveolar bone proper microstructure via hypoxia-inducible factor and VEGF expression in periodontal ligaments of growing rats. Front. Physiol. 7, 416. 10.3389/fphys.2016.00416 27695422 PMC5025444

[B36] Oishi S. Shimizu Y. Hosomichi J. Kuma Y. Nagai H. Maeda H. (2016a). Intermittent hypoxia induces disturbances in craniofacial growth and defects in craniofacial morphology. Arch. Oral Biol. 61, 115–124. 10.1016/j.archoralbio.2015.10.017 26552021

[B37] Pae E. K. Harper R. M. (2023). Intermittent hypoxia in neonatal rodents affects facial bone growth. PLoS One 18 (10), e0282937. 10.1371/journal.pone.0282937 37819881 PMC10566710

[B38] Pae E. K. Yoon A. J. Ahuja B. Lau G. W. Nguyen D. D. Kim Y. (2011). Perinatal intermittent hypoxia alters γ-aminobutyric acid: a receptor levels in rat cerebellum. Int. J. Dev. Neurosci. 29 (8), 819–826. 10.1016/j.ijdevneu.2011.09.003 21964325

[B39] Pien G. W. Pack A. I. Jackson N. Maislin G. Macones G. A. Schwab R. J. (2014). Risk factors for sleep-disordered breathing in pregnancy. Thorax 69 (4), 371–377. 10.1136/thoraxjnl-2012-202718 24262432 PMC6994201

[B40] Ren Z. Luo S. Cui J. Tang Y. Huang H. Ding G. (2023). Research progress of maternal metabolism on cardiac development and function in offspring. Nutrients 15 (15), 3388. 10.3390/nu15153388 37571325 PMC10420869

[B41] Ritchie H. E. Oakes D. J. Kennedy D. Polson J. W. (2017). Early gestational hypoxia and adverse developmental outcomes. Birth Defects Res. 109 (17), 1358–1376. 10.1002/bdr2.1136 29105381

[B42] Selvig K. A. Sorensen R. G. Wozney J. M. Wikesjö U. M. (2002). Bone repair following recombinant human bone morphogenetic protein-2 stimulated periodontal regeneration. J. Periodontol. 73 (9), 1020–1029. 10.1902/jop.2002.73.9.1020 12296587

[B43] Song R. Mishra J. S. Dangudubiyyam S. V. Baker T. L. Watters J. J. Kumar S. (2022). Gestational intermittent hypoxia programs hypertensive response in female rat offspring: impact of ovaries. J. Womens Health Dev. 5 (2), 185–196. 10.26502/fjwhd.2644-28840088 36337144 PMC9632646

[B44] Thompson L. P. Turan S. Aberdeen G. W. (2020). Sex differences and the effects of intrauterine hypoxia on growth and *in vivo* heart function of fetal Guinea pigs. Am. J. Physiol. Regul. Integr. Comp. Physiol. 319 (3), R243–R254. 10.1152/ajpregu.00249.2019 32639864 PMC7509254

[B45] Tomo S. Ogita M. Tomo I. (1997). Development of mandibular cartilages in the rat. Anat. Rec. 249 (2), 233–239. 10.1002/(SICI)1097-0185(199710)249:2<233::AID-AR10>3.0.CO;2-P 9335469

[B46] Tong W. Ganguly E. Villalobos-Labra R. Quon A. Spaans F. Giussani D. A. (2023). Sex-specific differences in the placental unfolded protein response in a rodent model of gestational hypoxia. Reprod. Sci. 30 (6), 1994–1997. 10.1007/s43032-022-01157-w 36574145 PMC10229681

[B47] Utting J. C. Robins S. P. Brandao-Burch A. Orriss I. R. Behar J. Arnett T. R. (2006). Hypoxia inhibits the growth, differentiation and bone-forming capacity of rat osteoblasts. Exp. Cell Res. 312 (10), 1693–1702. 10.1016/j.yexcr.2006.02.007 16529738

[B48] Vanderplow A. M. Kermath B. A. Bernhardt C. R. Gums K. T. Seablom E. N. Radcliff A. B. (2022). A feature of maternal sleep apnea during gestation causes autism-relevant neuronal and behavioral phenotypes in offspring. PLoS Biol. 20 (2), e3001502. 10.1371/journal.pbio.3001502 35113852 PMC8812875

[B49] Wang W. Tang J. Zhong M. Chen J. Li T. Dai Y. (2021). HIF-1 α may play a role in late pregnancy hypoxia-induced autism-like behaviors in offspring rats. Behav. Brain Res. 411, 113373. 10.1016/j.bbr.2021.113373 34048873

[B50] Wilson E. N. Mabry S. Bradshaw J. L. Gardner J. J. Rybalchenko N. Engelland R. (2022). Gestational hypoxia in late pregnancy differentially programs subcortical brain maturation in male and female rat offspring. Biol. Sex. Differ. 13 (1), 54. 10.1186/s13293-022-00463-x 36175941 PMC9524087

[B51] Wilson R. L. Stephens K. K. Jones H. N. (2023). Placental nanoparticle gene therapy normalizes gene expression changes in the fetal liver associated with fetal growth restriction in a fetal sex-specific manner. J. Dev. Orig. Health Dis. 14 (3), 325–332. 10.1017/S2040174423000016 36794386 PMC10947591

[B52] Wongkitikamjorn W. Hosomichi J. Wada E. Maeda H. Satrawaha S. Hong H. (2022). Gestational intermittent hypoxia induces mitochondrial impairment in the geniohyoid muscle of offspring rats. Cureus 14 (5), e25088. 10.7759/cureus.25088 35600069 PMC9117862

[B53] Wongkitikamjorn W. Wada E. Hosomichi J. Maeda H. Satrawaha S. Hong H. (2023). Metabolic dysregulation and decreased capillarization in skeletal muscles of male adolescent offspring rats exposed to gestational intermittent hypoxia. Front. Physiol. 14, 1067683. 10.3389/fphys.2023.1067683 36711021 PMC9878705

[B54] Yamaki Y. Tsuchikawa K. Nagasawa T. Hiroyasu K. (2005). Embryological study of the development of the rat temporomandibular joint: highlighting the development of the glenoid fossa. Odontology 93 (1), 30–34. 10.1007/s10266-005-0046-9 16170473

