# Supplementary material for: Gestational intermittent hypoxia reduces mandibular growth with decreased Sox9 expression and increased Hif1a expression in male offspring rats
Source: Front Physiol. 2024 Jun 11;15:1397262. doi: 10.3389/fphys.2024.1397262 (PMC11196756; doi:10.3389/fphys.2024.1397262)
Supplement: Supplementary file 1 [file DataSheet1.docx]

Supplementary Material

Gestational intermittent hypoxia reduces mandibular growth with decreased Sox9 expression and increased Hif1a expression in male offspring rats

Takumi Suzuki^1,2^, Jun Hosomichi^1,2*^, Hideyuki Maeda^3^, Yuji Ishida^1^, Risa Usumi-Fujita^1^, Manaka Moro^1^, Korkuan Jariyatheerawong^1,4^, Takashi Ono^1^

*** Correspondence:**Jun Hosomichi
[hosomichi.orts@tmd.ac.jp](mailto:hosomichi.orts@tmd.ac.jp)


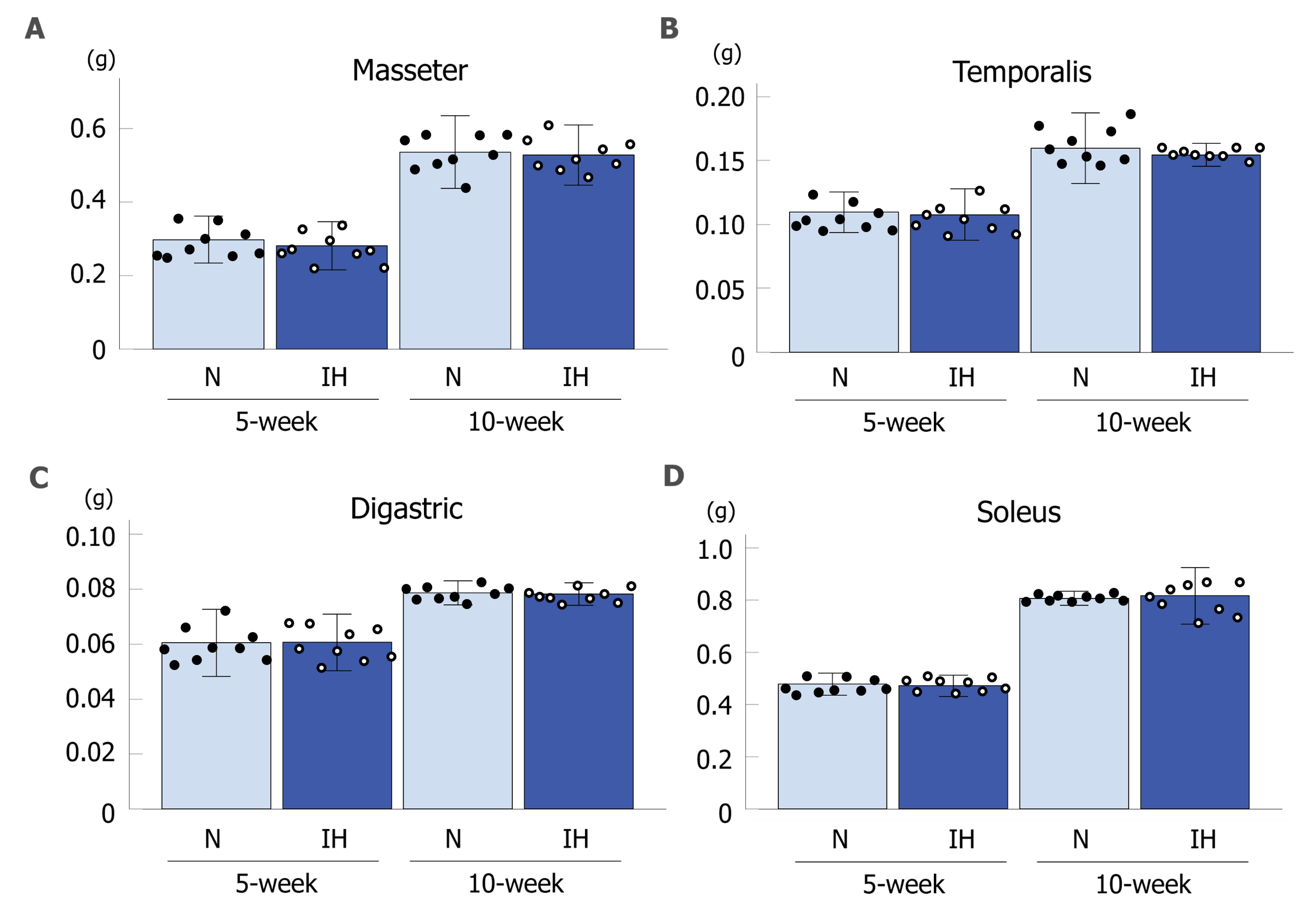


**Supplementary Figure 1.** Skeletal muscle weight of male rats. Comparison of changes in masseter (*A*), temporalis (*B*), digastric (*C*) and soleus (*D*) muscles. *: p < 0.05. Abbreviations: IH, intermittent hypoxia; N, normoxic; 5-week, 5 weeks of age; 10-week, 10 weeks of age.

**
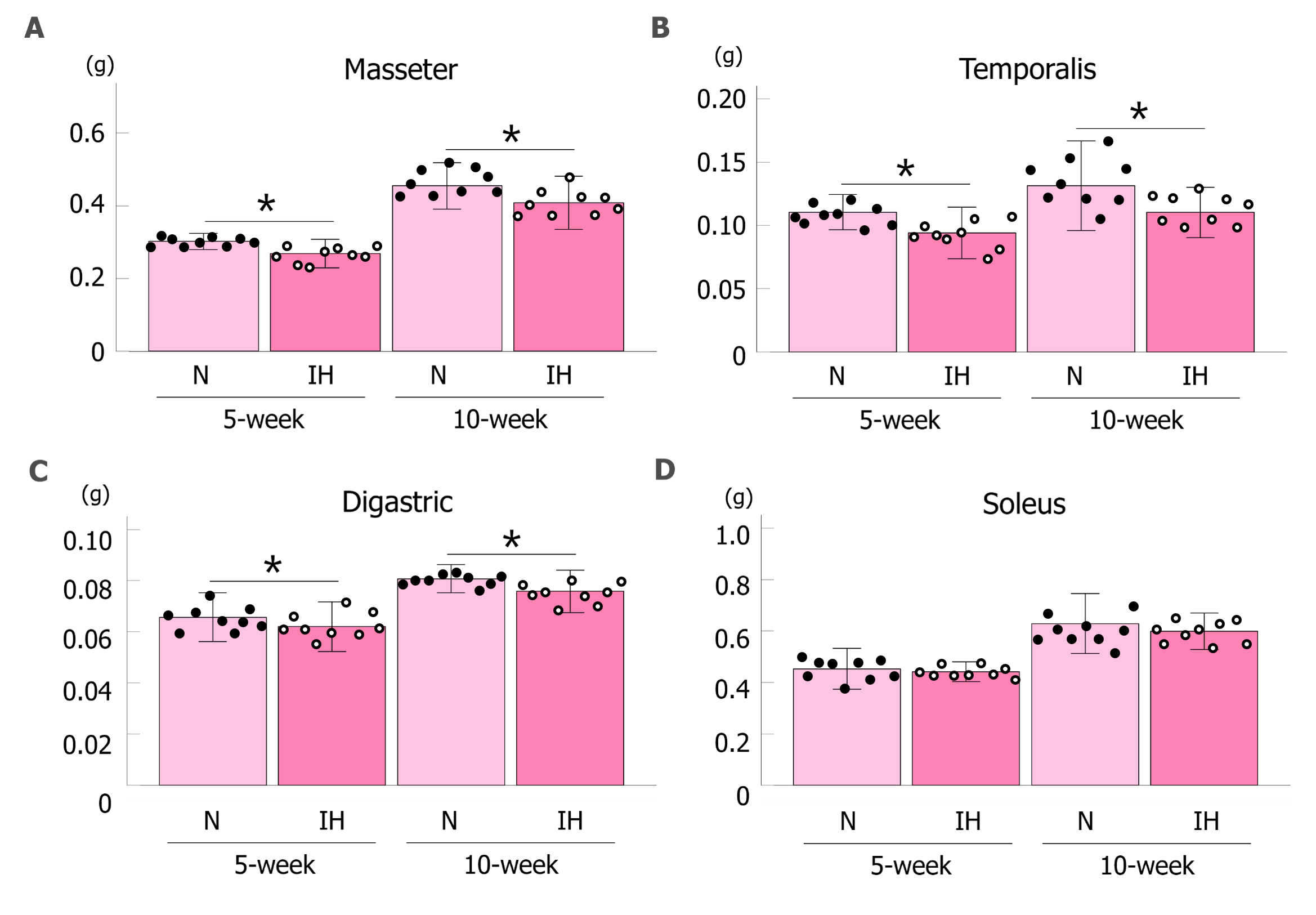
**

**Supplementary Figure 2.** Skeletal muscle weight of female rats. Comparison of changes in masseter (*A*), temporalis (*B*), digastric (*C*) and soleus (*D*) muscles. *: p < 0.05. Abbreviations: IH, intermittent hypoxia; N, normoxic; 5-week, 5 weeks of age; 10-week, 10 weeks of age.
